# A Comparative Study on Improved Arrhenius-Type and Artificial Neural Network Models to Predict High-Temperature Flow Behaviors in 20MnNiMo Alloy

**DOI:** 10.1155/2014/108492

**Published:** 2014-02-12

**Authors:** Guo-zheng Quan, Chun-tang Yu, Ying-ying Liu, Yu-feng Xia

**Affiliations:** School of Material Science and Engineering, Chongqing University, Chongqing 400044, China

## Abstract

The stress-strain data of 20MnNiMo alloy were collected from a series of hot compressions on Gleeble-1500 thermal-mechanical simulator in the temperature range of 1173**∼**1473 K and strain rate range of 0.01**∼**10 s^−1^. Based on the experimental data, the improved Arrhenius-type constitutive model and the artificial neural network (ANN) model were established to predict the high temperature flow stress of as-cast 20MnNiMo alloy. The accuracy and reliability of the improved Arrhenius-type model and the trained ANN model were further evaluated in terms of the correlation coefficient (*R*), the average absolute relative error (AARE), and the relative error (*η*). For the former, *R* and AARE were found to be 0.9954 and 5.26%, respectively, while, for the latter, 0.9997 and 1.02%, respectively. The relative errors (*η*) of the improved Arrhenius-type model and the ANN model were, respectively, in the range of −39.99%**∼**35.05% and −3.77%**∼**16.74%. As for the former, only 16.3% of the test data set possesses *η*-values within ±1%, while, as for the latter, more than 79% possesses. The results indicate that the ANN model presents a higher predictable ability than the improved Arrhenius-type constitutive model.

## 1. Introduction

20MnNiMo is a low carbon steel with moderate strength, superior plasticity and toughness, good ductility and workability. Due to its low neutron irradiation sensitivity and superior performance, 20MnNiMo is increasingly and extensively applied in manufacture of large and medium-sized nuclear reactor pressure vessel [1]. The understanding of the flow behaviors of metals and alloys at a hot deformation condition has a great importance for designers engaged in metal forming (hot rolling, forging, extrusion, etc.), since flow behaviors have an effective role on material deformation pattern as well as on the kinetics of metallurgical transformation. The constitutive relationships are often used to describe the material deformation pattern in a form that can be used in computer codes to model the forging response of mechanical part under the prevailing loading conditions [2]. So far, Lee et al. [3] have investigated the influence of composition and distribution of carbides on the fracture toughness of 20MnNiMo steel. Sun and his colleagues describe the mechanical responses and microstructural evolutions of the 20MnNiMo steel under various hot deformation conditions [4]. Nevertheless, there is still a lack of basic understanding of the hot deformation behavior of 20MnNiMo alloys until now [1, 3, 4]. Therefore, it is necessary to investigate high temperature flow behavior of 20MnNiMo alloy and accurately predict the stress.

Constitutive relationship is a mathematical representation for describing the correlation between flow stress, strain, strain rate and temperature in a wide range of working conditions [5, 6]. Many researchers have attempted to develop constitutive equations from the experimental data to accurately describe the thermal deformation behavior of materials [7–11]. And constitutive model expressed by the hyperbolic sine law has been extensively applied to describe the hot deformation behavior of materials. Various modifications of this model have also been suggested to improve its predictability [8–13]. Slooff et al. [11] introduced strain-dependent parameters in the hyperbolic sine constitutive model to predict the flow stress in a wrought magnesium alloy. Lin et al. [12] developed the strain-dependent hyperbolic sine constitutive model by compensation of strain rate in Zener-Hollomon parameter $\left(Z=\dot{\varepsilon }\exp(Q/RT)\right)$ to predict flow behavior of 42CrMo steel, and the results showed that the strain-compensated Arrhenius-type equations could track the deformation behavior more accurately than the other equations. Later, an improved Arrhenius-type constitutive equation incorporating a series of polynomial functions for each coefficient followed by lin's method was constructed to describe the high temperature flow behaviors of AZ81 magnesium alloy [13].

However, the improved Arrhenius-type constitutive model also has its own drawbacks in some certain cases such as low accuracy for predicting the relationships between flow stresses and processing variables and poor adaptability for the new experimental data [13, 14]. Recently, the artificial neural network (ANN) as an artificial intelligent approach was introduced to establish the constitutive relationship and further model the flow behaviors under hot compression of many metals and alloys [15–17]. Owing to its inherently high parallelism, ANN is ideally suited for the problem of estimating the flow stress from the available experimental data [16, 17]. It is in particular suitable for treating complex and nonlinear relationships and has been successfully applied to the prediction of constitutive relationships for some alloys [18]. More importantly, it is quite convenient to present the deformation behavior of materials with favorable accuracy under hot working conditions [19]. Xiao et al. [20] make a comparative study on the Arrhenius-type constitutive model considering strain compensation and artificial neural network models to predict the hot deformation behavior of the 12Cr3WV steel, and the results showed that the ANN model can predict the flow stress more efficient and accurate than the Arrhenius-type constitutive equations.

The objective of this investigation is to make a comparative study on the improved Arrhenius-type Constitutive model and artificial neural network models on their capability to predict the high-temperature flow behavior of 20MnNiMo alloy. Experimental data from isothermal hot compression tests on Gleeble-1500 thermal-mechanical simulator in a temperature range from 1173 K to 1473 K and a strain rate range from 0.01 s^−1^ to 10 s^−1^ are used to resolve the improved Arrhenius-type Constitutive model and to develop artificial neural network model. Subsequently, the suitability of these models to predict the elevated temperature flow behavior was evaluated based on the correlation coefficient (*R*), average absolute relative error (AARE), and relative error (*η*).

## 2. Materials and Experiment Procedures

The chemical compositions (wt%) of 20MnNiMo alloy were C-0.2, Si-0.16, Mn-1.53, S-0.0022, P-0.0063, Ni-0.81, Mo-0.57, Cr-0.14, V-0.005, Cu-0.04, and Fe (balance). Before the experiment, the extruded rod was homogenized under temperature 1523 K for 12 h. Then the rod was scalped to height 12 mm and diameter 10 mm with grooves on both sides filled with machine oil mingled with graphite powder as lubricant to reduce friction between the anvils and specimen. On a computer-controlled, servohydraulic Gleeble-1500 machine, the specimens were resistance-heated at a heating rate of 10 K/s and held at a certain temperature for 180 s to ensure a uniform starting temperature and decrease the material anisotropy. Sixteen specimens were compressed with a height reduction of 60% at four different temperatures of 1173 K, 1273 K, 1373 K, and 1473 K and four different strain rates of 0.01 s^−1^, 0.1 s^−1^, 1 s^−1^, and 10 s^−1^. During the compressing process the variations of stress and strain were monitored continuously by a personal computer equipped with an automatic data acquisition system. The true stress-strain relationships were derived from the nominal stress-strain curves collected according to the following formula: *σ*_*T*_ = |*σ*_*N*_(1 + *ε*_*N*_)|, *ε*_*T*_ = |ln⁡(1 + *ε*_*N*_)|, where *σ*_*T*_ is the true stress, *σ*_*N*_ is the nominal stress, *ε*_*T*_ is the true strain, and *ε*_*N*_ is the nominal strain.

## 3. Results and Discussion 

### 3.1. Flow Stress Behavior

**Figure 1:**
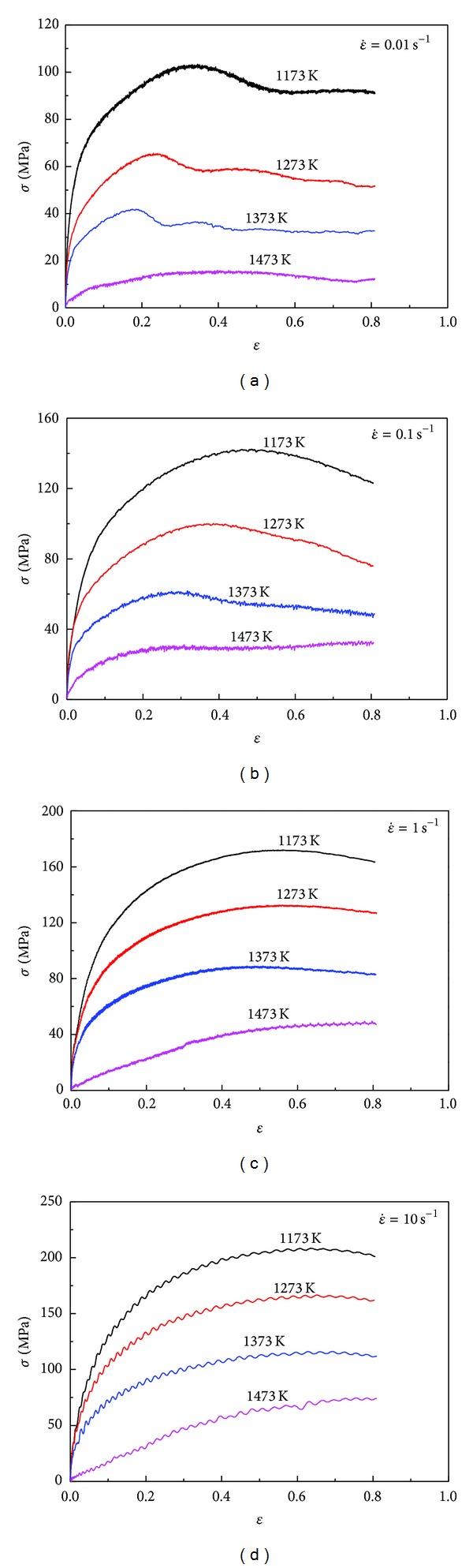
The true stress-strain curves of as-cast 20MnNiMo alloy under the different temperatures with strain rates (a) 0.01 s^−1^, (b) 0.1 s^−1^, (c) 1 s^−1^, (d) 10 s^−1^.

The true stress-strain curves of 20MnNiMo alloy compressed at different deformation conditions are shown in Figures 1(a), 1(b), 1(c), and 1(d). The flow stress as well as the shape of the flow curves is sensitively dependent on strain rate and temperature. Comparing these curves with one another, it is found that, for a specific strain rate, the flow stress decreases markedly with temperature, while at a certain temperature, the flow stress generally increases as the strain rate increases due to an increase of dislocation density and the dislocation multiplication rate.

From the true stress-strain curves in Figures 1(a), 1(b), 1(c), and 1(d), it can be seen that the stress evolution with strain exhibits three distinct stages [21]. At the first stage, where work hardening (WH) predominates and causes dislocations to polygonize into stable subgrains, flow stress exhibits a rapid increase to a critical value with increasing strain, resulting in equiaxed DRX grains. At the second stage, flow stress exhibits a smaller and smaller increase until a peak value or an inflection of work-hardening rate, which shows that the thermal softening due to DRX and dynamic recovery (DRV) becomes more and more predominant, then it exceeds WH. At the third stage, three types of curve variation tendency can be generalized as follows: decreasing gradually to a steady state with DRX softening (1173~1473 K and 0.01 s^−1^, 1373~1473 K, and 0.1 s^−1^), maintaining higher stress level without significant softening and work-hardening (1173~1273 K and 0.1 s^−1^, 1173~1373 K and 1 s^−1^, 1173~1373 K and 10 s^−1^), and increasing continuously with significant work hardening (1473 K and 1 s^−1^, 1473 K and 10 s^−1^). Thus, it can be concluded that the typical form of flow curve with DRX softening, including a single peak followed by a steady state flow as a plateau, is more recognizable at higher temperatures and lower strain rates. That is because at lower strain rates and higher temperatures, the higher DRX softening rate slows down the rate of work-hardening, and both the peak stress and the onset of steady state flow are therefore shifted to lower strain levels [22–27].

### 3.2. Improved Arrhenius-Type Constitutive Model

In order to further investigate the thermal deformation behaviors of 20MnNiMo alloy, it is necessary to study the constitutive characteristics. The stress-strain data obtained from hot compression tests can be used to determine the material constants of the constitution equation. The Arrhenius equation is widely used to describe the relationship between flow stress, deformation temperature, and strain rate, especially at high temperature [28]. Furthermore, the effects of deformation temperature and strain rate on the deformation behaviors can be represented by Zener-Hollomon parameter (*Z*) in an exponential equation [29]. The two equations are mathematically expressed as

(1) $$\begin{array}{c} Z=\dot{\varepsilon }\exp\left(Q/RT\right). \end{array}$$

(2) $$\begin{array}{c} \dot{\varepsilon }=AF\left(\sigma \right)\exp\left(-Q/RT\right), \end{array}$$

where,

(3) $$\begin{array}{c} F\left(\sigma \right)=\{\begin{array}{cc} \sigma^{n} & \alpha \sigma <0.8\\ \exp\left(\beta \sigma \right) & \alpha \sigma >1.2\\ {\left[\sinh\left(\alpha \sigma \right)\right]}^{n} & \;\text{for}\;\text{all}\;\sigma \end{array} \end{array}$$

in which $\dot{\varepsilon }$ is the strain rate (s^−1^), *R* is the universal gas constant (8.31 J·mol^−1^·K^−1^), *T* is the absolute temperature (K), *Q* is the activation energy of DRX (kJ·mol^−1^), *σ* is the flow stress (MPa) for a given stain, *A*, and *α* and *n* are the material constants, *α* = *β*/*n*.

It is commonly accepted that the effect of strain on stress has not been considered in (1) and (2). Here the effects of deformation strain on stress are investigated by the consideration of the influence of strain on a series of variable coefficients (including activation energy of deformation *Q*, material constants *n* and *α*, and structure factor *A*) in Arrhenius type model. The following is taking the strain of 0.2 as an example.

For the low stress level (*ασ* < 0.8) and high stress level (*ασ* > 1.2), by substituting power law and exponential law of *F*(*σ*) into (2), the relationships between flow stress and strain rate can be expressed in the following equations, respectively,

(4) $$\begin{array}{c} \dot{\varepsilon }=B\sigma^{n}. \end{array}$$

(5) $$\begin{array}{c} \dot{\varepsilon }=B^{\prime }\exp\left(\beta \sigma \right), \end{array}$$

where *B* and *B*′are the material constants that are dependent of deformation temperatures. Taking natural logarithms on both sides of (4) and (5), respectively, gives

(6) $$\begin{array}{c} \ln\sigma =\frac{1}{\beta }\ln\dot{\varepsilon }-\frac{1}{\beta }B^{\prime }, \end{array}$$

(7) $$\begin{array}{c} \sigma =\frac{1}{\beta }\ln\dot{\varepsilon }-\frac{1}{\beta }B^{\prime }. \end{array}$$

**Figure 2:**
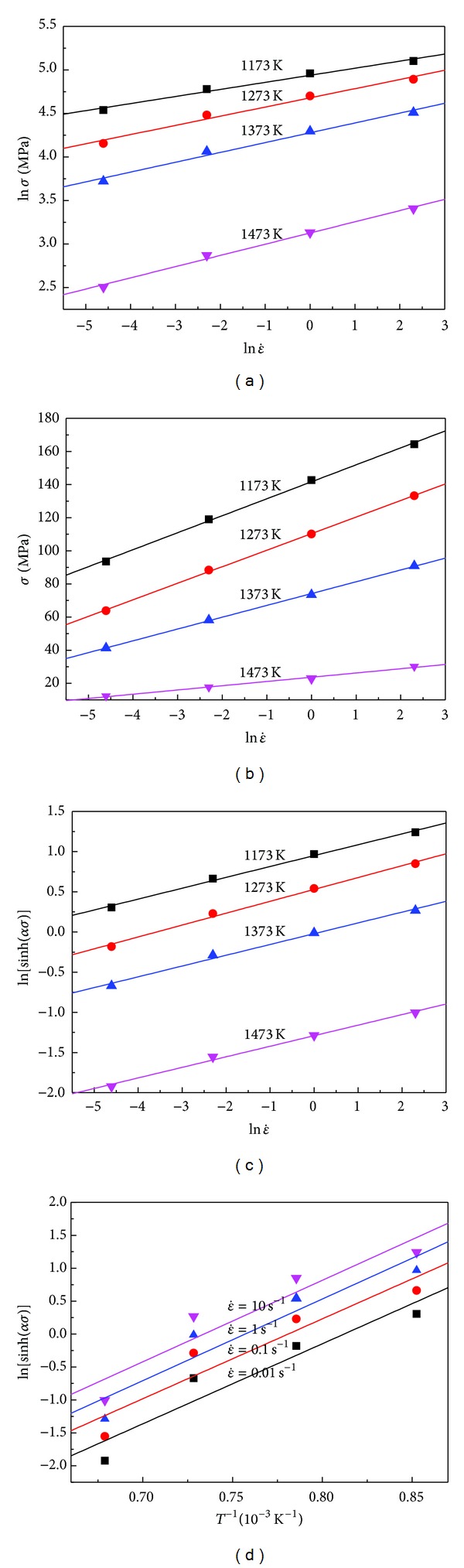
The relationships between (a) ln⁡*σ* and $\ln\dot{\varepsilon }$, (b) *σ* and $\ln\dot{\varepsilon }$, (c) ln⁡[sinh⁡(*ασ*)] and *T* ^−1^, and (d) ln⁡[sinh⁡(*ασ*)] and $\ln\dot{\varepsilon }$.

Then, $1/n=\text{d}\ln\sigma /\text{dln}\;\dot{\varepsilon }$ and $1/\beta =\text{d}\sigma /\text{d}\ln\dot{\varepsilon }$. Substituting the values of the flow stress and corresponding strain rate at the strain of 0.2 into the logarithms (6) and (7) gives the relationship between stress and strain rate as shown in Figure 2. Figures 2(a) and 2(b) show the relationships of $\sigma -\ln\dot{\varepsilon }$ and $\ln\sigma -\ln\dot{\varepsilon }$ for *ε* = 0.2 at the temperatures of 1173 K, 1273 K, 1373 K, and 1473 K. In Figure 2(a) the average value of all the lines' slopes is equal to the inverse of *n*-value, thus *n* = 7.30547 MPa^−1^ for *ε* = 0.2. Meanwhile in Figure 2(b) the average value of all the lines' slopes is equal to the inverse of *β*-value, thus *β* = 0.098854 MPa^−1^ for *ε* = 0.2. Then *α* = *β*/*n* = 0.0011897.

For all the stress level (including low and high-stress levels), (2) can be represented as follows:

(8) $$\begin{array}{c} \dot{\varepsilon }=A{\left[\sinh\left(\alpha \sigma \right)\right]}^{n}\exp\left(-Q/RT\right). \end{array}$$

By substituting Zener-Hollomon parameter $Z=\dot{\varepsilon }\exp(Q/RT)$ into (8), the flow stress can be expressed as follows (9):

(9) $$\begin{array}{c} \sigma =\frac{1}{\alpha }\ln\left\{{\left(\frac{Z}{A}\right)}^{1/n}+{\left[{\left(\frac{Z}{A}\right)}^{2/n}+1\right]}^{1/2}\right\}. \end{array}$$

Taking the logarithm of both sides of (8) gives

(10) $$\begin{array}{c} \ln\sinh\left(\alpha \sigma \right)=\frac{1}{n}\ln\dot{\varepsilon }+\frac{Q}{nRT}-\frac{1}{n}\ln A. \end{array}$$

For the fixed temperature and strain, by differentiating (10), the value of *n* can be expressed as

(11) $$\begin{array}{c} \frac{1}{n}=\frac{\text{d}\ln\sinh\left(\alpha \sigma \right)}{\text{d}\;\ln\dot{\varepsilon }}. \end{array}$$

The value of *n* can be obtained from the slope in a plot of $\ln\sinh(\alpha \sigma )-\ln\dot{\varepsilon }$ by substituting the values of the flow stress and strain rate for all the tested temperatures into (11) and the linear relationships between ln⁡⁡sinh⁡(*ασ*) and $\ln\dot{\varepsilon }$ at different temperatures were fitted out as Figure 2(c). The mean value of all the intercepts of ln⁡⁡sinh⁡(*ασ*) versus $\ln\dot{\varepsilon }$ plots is accepted as *A* value; furthermore *A* value for *ε* = 0.2 is obtained as 3.769 × 1012 s^−1^.

For the given strain rate conditions, differentiating (10) gives

(12) $$\begin{array}{c} Q=Rn\left\{\frac{\text{d}\left[\ln\;\sinh\left(\alpha \sigma \right)\right]}{\text{d}\left(1/T\right)}\right\}. \end{array}$$

It is clear that the value of *Q* can be derived from the slope in a plot of ln⁡[sinh⁡⁡(*α*|*σ*|)] as a function of 1/*T* as shown in Figure 2(d). From a group of parallel and straight lines in Figure 2(d), the value of activated energy (*Q*) as 744.34 kJ/mol can be easily evaluated by averaging the values of (*Q*) under different strain rates.

**Figure 3:**
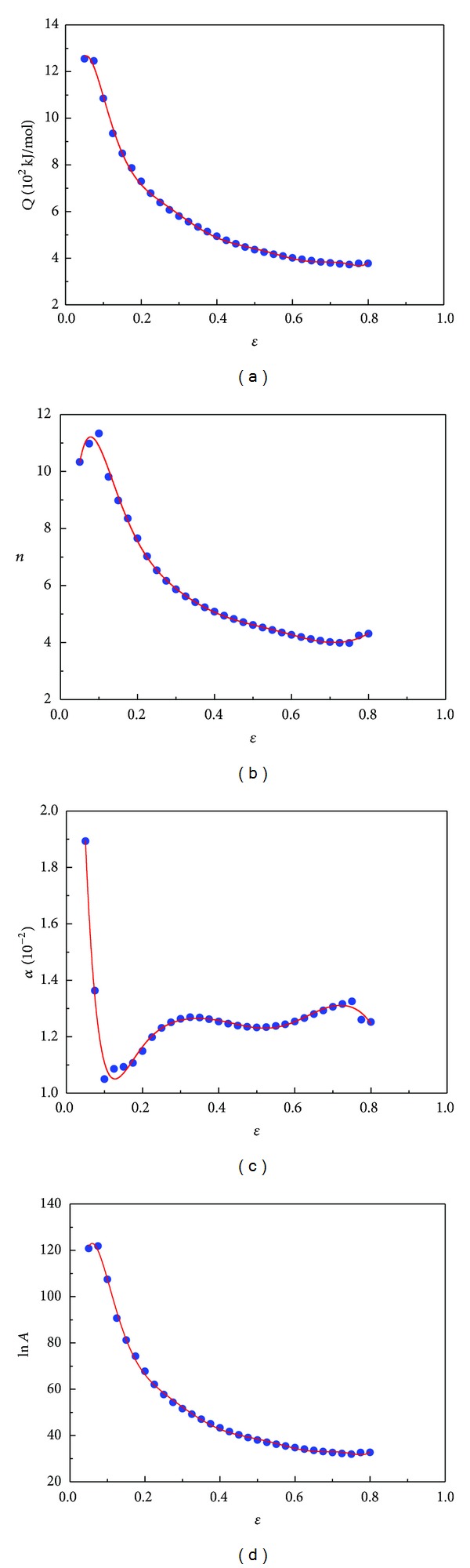
Relationship between (a) *Q*, (b) *n*, (c) *α*, (d) ln⁡⁡*A* and true strain by polynomial fit of 20MnNiMo alloy.

Then, the values of material constants (*Q*,*n*, ln⁡*A*, and *α*) of the constitutive equations were computed under different deformation strains within the range of 0.05~0.80 and the interval of 0.025. The relationships between, *Q*,*n*, ln⁡*A*, *α*, and true strain for 20MnNiMo alloy (Figure 3) can be polynomially fitted by the compensation of strain, as shown in (13). The polynomial fit results of *Q*,*n*, ln⁡*A*, and *α* of 20MnNiMo alloy are provided in Table 1. Consider the following:

(13) Q=j(ε)=B0+B1ε+B2ε2+B3ε3+B4ε4 +B5ε5+B6ε6+B7ε7+B8ε8+B9ε9n=h(ε)=C0+C1ε+C2ε2+C3ε3+C4ε4 +C5ε5+C6ε6+C7ε7+C8ε8+C9ε9ln⁡⁡A=ln⁡f(ε)=D0+D1ε+D2ε2+D3ε3 +D4ε4+D5ε5+D6ε6+D7ε7+D8ε8+D9ε9α=g(ε)=E0+E1ε+E2ε2+E3ε3+E4ε4 +E5ε5+E6ε6+E7ε7+E8ε8+E9ε9.

Substituting *Q*,*n*,  ln⁡*A*, and *α* in (14) into (8) gives the relationships between $\dot{\varepsilon }$,*T*, and *σ* as follows (14):

(14) $$\begin{array}{c} \left|\dot{\varepsilon }\right|=f\left(\varepsilon \right){\left\{\sinh\left[g\left(\varepsilon \right)\left|\sigma \right|\right]\right\}}^{h(\varepsilon )}\times \exp\left(\frac{j\left(\varepsilon \right)}{8.31T}\right). \end{array}$$

Thus, $Z=\left|\dot{\varepsilon }\right|\exp\left[j(\varepsilon )/8.31T\right]$. Furthermore, the constitutive equation for flow behavior of 20MnNiMo alloy in a wide strain range of 0.05~0.80 can be expressed as follows (15):

(15) |σ|=1g(ε)ln⁡{(|ε˙|exp⁡[j(ε)/8.314T]f(ε))1/h(ε)     +[(|ε˙|exp⁡[j(ε)/8.314T]f(ε))2/h(ε)+1]1/2},

where *j*(*ε*),*h*(*ε*), *f*(*ε*), and *g*(*ε*) are the polynomial functions of *Q*,*n*,*A*, and *α* at different true strains, and their expressions are as (13) and Table 1.

### 3.3. Artificial Neural Model

**Figure 4:**
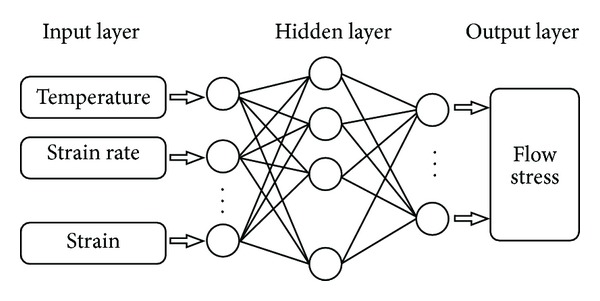
Schematic illustration of the neural network architecture.

Artificial neural network (ANN) is a powerful treatment system for data information, which can mimic complex and nonlinear relationships through the application of many nonlinear processing units called neurons. A typical artificial neural networks architecture consists of an input layer, an output layer, and a hidden layer. Some neuron serves the input variables, some provide the output, and the rest of the neurons remain hidden [30]. Back-propagation algorithm is the typical means of adjusting the weights and biases by utilizing gradient descent to minimize the target error, which is approximated in the vector space created by the weights and biases [31, 32]. Hence, a three-layer feed forward back-propagation (BP) artificial neural network (as shown in Figure 4) was employed to predict the hot deformation behavior of 20MnNiMo alloy in present work. The inputs of the ANN model are strain, log strain rate, and temperature. The output of the ANN model is flow stress.

When developing the ANN model, 396 random data sets from the true stress-true strain curves were used to train the network model, and 100 data sets at true strain between 0.05 and 0.80 with interval of 0.025 were applied to test the predictability of the ANN model. Before training the network, both input and output variable datasets were scaled between 0 and 1 in order to ensure that each variable lay in the same range during the training and the testing. The following equation was used widely for unification of data*T*, *ε*, and *σ* [25]:

(16) $$\begin{array}{c} X^{\prime }=\frac{X-0.95X_{\min}}{1.05X_{\max}-0.95X_{\min}}, \end{array}$$

where *X* is the original data, *X*_min⁡_ and *X*_max⁡_ are the minimum and maximum value of *X*, respectively, and *X*′ is the unified data of the corresponding *X*. Since the $\dot{\varepsilon }$ changed sharply and the minimum value of $\dot{\varepsilon }$ after unification was too small for the ANN model to learn, the following equation is developed to unify the value of $\dot{\varepsilon }$:

(17) $$\begin{array}{c} {\dot{\varepsilon }}^{\prime }=\frac{\left(3\;+\text{lg}\;\dot{\varepsilon }\;\right)-0.95\left(3+\text{lg}\;{\dot{\varepsilon }}_{\min}\right)}{1.05\left(3+\text{lg}\;{\dot{\varepsilon }}_{\max}\right)-0.95\left(3+\text{lg}\;{\dot{\varepsilon }}_{\min}\right)} \end{array}$$

in which a constant 3 is defined to make the unified data be positive [25]. The transfer functions were “tan sigmoid”, and the training function was “Trainlm”.

**Figure 5:**
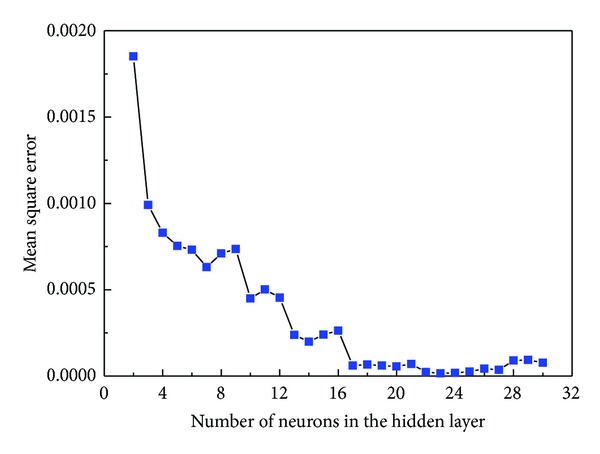
Influence of hidden neutrons on the network performance.

In order to determine the appropriate number of neurons in the hidden layer, the trial-and-error procedure was started with two neurons in the hidden layer and further carried out with more neurons.  Figure 5 shows the influence of number of neurons in the hidden layer on the network performance. The value of mean square error is applied to check the ability of a particular architecture. It is founded that the value of mean square error decreases to the minimum value when the number of neurons is 23, which indicates that the network with 23 hidden neurons was the optimal structure for the prediction of flow stress of 20MnNiMo alloy.

**Figure 6:**
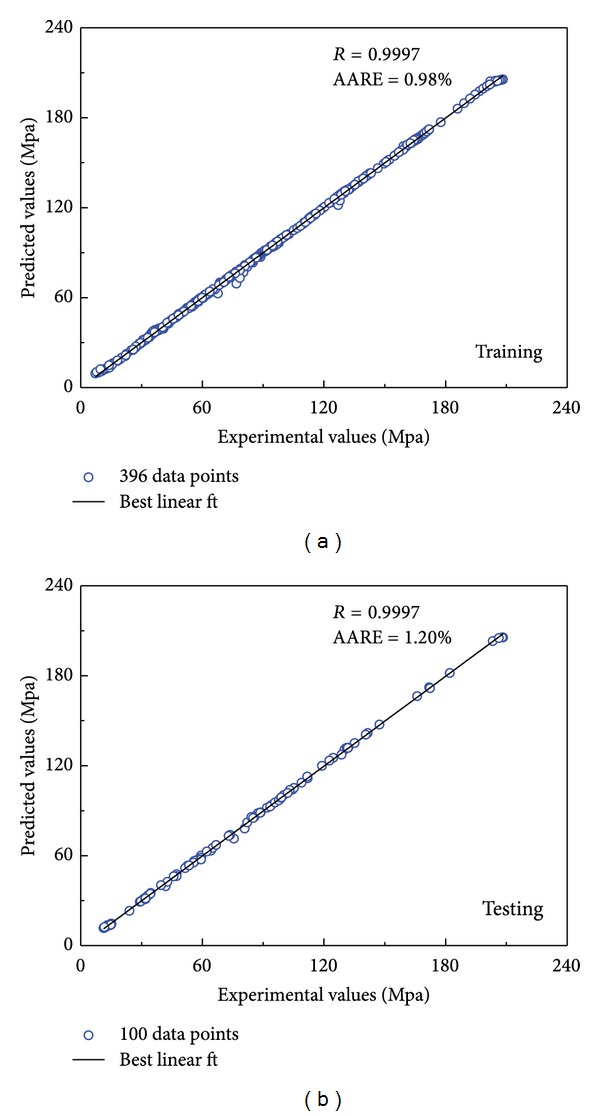
Correlation between the experimental and ANN predicted data for the (a) training and (b) test data.

Meanwhile, an evaluator, correlation coefficient (*R*) and average absolute relative error (AARE) are introduced in training and testing datasets to evaluate the performance of the ANN training work. These are defined as follows [18]:

(18) $$\begin{array}{c} R=\frac{\sum_{i=1}^{N}\left(E_{i}-\overset{-}{E}\right)\left(P_{i}-\overset{-}{P}\right)}{\sqrt{\sum_{i=1}^{N}{\left(E_{i}-\overset{-}{E}\right)}^{2}\sum_{i=1}^{N}{\left(P_{i}-\overset{-}{P}\right)}^{2}}}\\ \text{AARE}\left(\%\right)=\frac{1}{N}\overset{N}{\underset{i=1}{\sum }}\left|\frac{E_{i}-P_{i}}{E_{i}}\right|\times 100, \end{array}$$

where *E* is the sample of experimental value, *P* is the sample of predicted value by ANN model, $\overset{-}{E}$ and $\overset{-}{P}$ are the mean value of *E* and *P*, respectively, and *N* is the number of strain-stress samples. Comparisons of ANN with 23 hidden neurons predicted flow stress with experimental ones during training and testing are shown in Figures 6(a) and 6(b). Standard statistical performance indices of the ANN model during training, *R* and AARE, are 0.9997 and 0.98%; and those of test, *R* and AARE, are 0.9997 and 1.02%, respectively. These observations indicate that the trained ANN model with 23 hidden neurons has good capability to predict and generalize the hot deformation behavior of 20MnNiMo alloy.

### 3.4. Comparative Evaluation of the Improved Arrhenius-Type Constitutive Equations and the ANN Model

**Figure 7:**
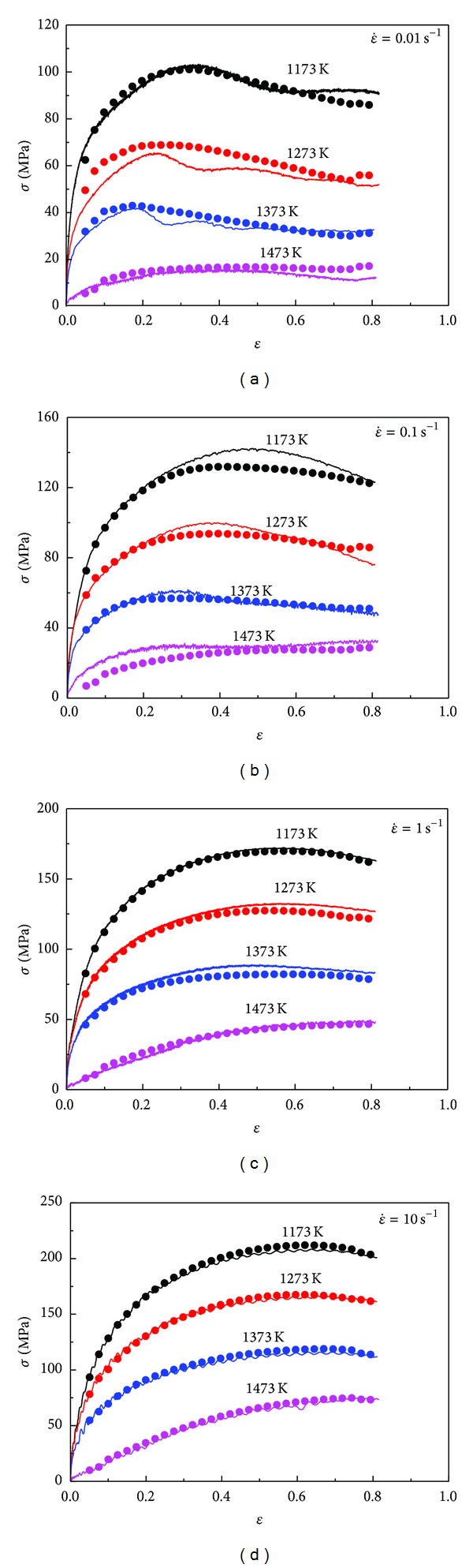
Comparison between the experimental and predicted flow stress by improved Arrhenius-type constitutive model at different strain rates and temperatures (a) 0.01 s^−1^, (b) 0.1 s^−1^, (c) 1 s^−1^, and (d) 10 s^−1^.

Figure 7 shows comparisons of the experimental flow stress data with values calculated by improved Arrhenius-type model for the four different temperatures under strain rates of 0.01 s^−1^, 0.1 s^−1^, 1 s^−1^, and 10 s^−1^. It can be easily found that the case of high strain rate of 1 s^−1^ and 10 s^−1^ is the best ideal one, and the experimental and predicted results are in good agreement. However, for the case of low strain rates of 0.01 s^−1^ and 0.1 s^−1^, there are obvious errors between the experimental and predicted results. The predicted flow stress is larger than the experimental one for the temperatures of 1273 K, 1373 K, and 1473 K at the strain rate of 0.01 s^−1^, while the contrary conclusion will be obtained for the temperature of 1173 K and 1473 K at the strain rate of 0.1 s^−1^. Furthermore, the predicted flow stress can not accurately describe the DRX characteristic very well.

**Figure 8:**
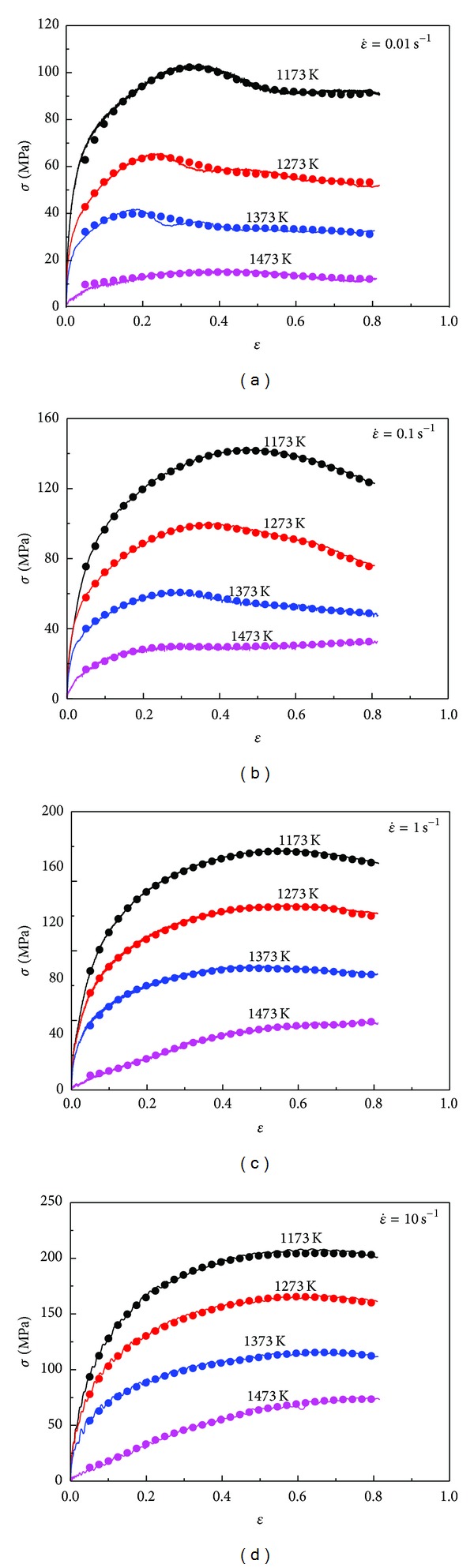
Comparison between the experimental and predicted flow stress by ANN model at different strain rates and temperatures (a) 0.01 s^−1^, (b) 0.1 s^−1^, (c) 1 s^−1^, and (d) 10 s^−1^.

The predicted flow stresses from ANN model and corresponding experimental ones at different temperatures and strain rates are compared as shown in Figure 8. It could be observed that the predicted values can track the experimental results very well throughout both the work hardening stage and dynamic softening stage (whatever it is DRX or DRV softening mechanism) in a wide temperature range of 1173~1473 K, a wide strain rate range of 0.01~10 s^−1^, and a wide strain range of 0.05~0.8. All above suggests that the present ANN model has an excellent capability and high accuracy to describe the flow behavior of 20MnNiMo at different temperature and strain rates.

**Figure 9:**
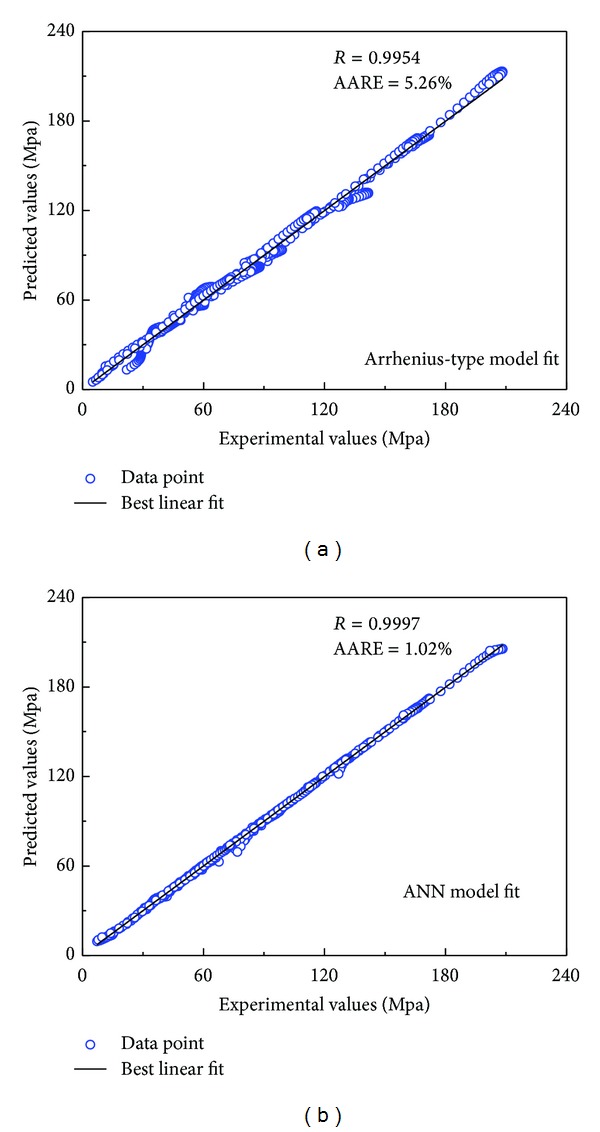
Correlation between experimental and predicted flow stress data from (a) constitutive model and (b) ANN model.

The accuracy of the improved Arrhenius-type constitutive equations and the ANN model was further quantified by the correlation coefficient (*R*) and the average absolute relative error (AARE). They can be expressed as (18).  Figure 9 shows the plots of experimental values and predicted values predicted by improved Arrhenius-type constitutive equations and the ANN model, respectively. It is clearly seen that most of the data points lie very close to the line, and the correlation coefficients (*R*) for the Improved Arrhenius-type and ANN models are 0.9954 and 0.9997, respectively. The average absolute relative error (AARE) of the Improved Arrhenius-type model is 5.26%, which is larger than the value 1.02% of the ANN model.

**Figure 10:**
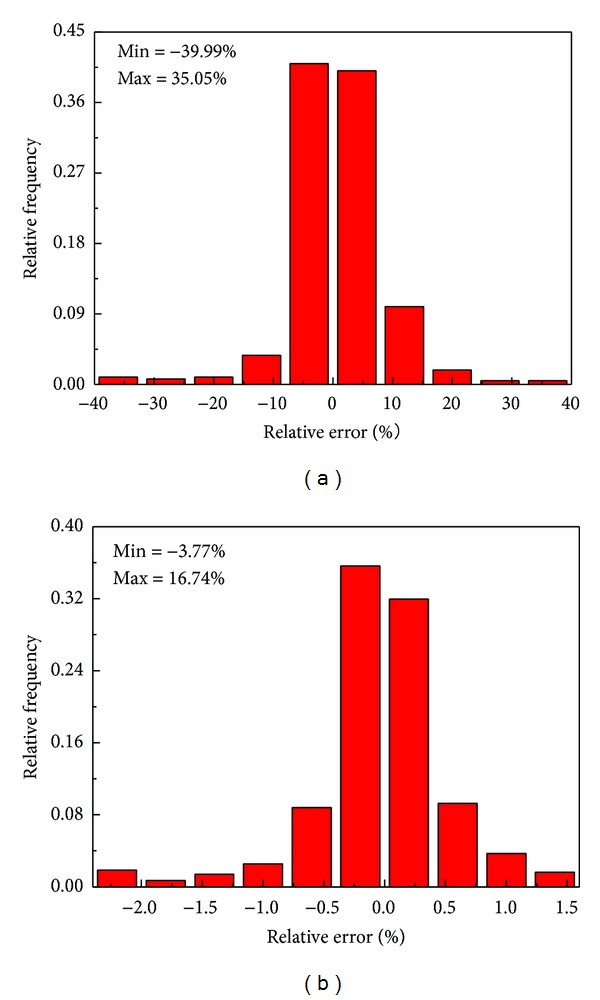
Distributions on prediction error by (a) constitutive model and (b) ANN model.

The accuracy of the improved Arrhenius-type constitutive equations and the ANN model was further investigated by statistical analysis of the relative errors (*η*) between the experimental values and predicted values, which were calculated by (19) as follows:

(19) $$\begin{array}{c} \eta \left(\%\right)=\frac{P_{i}-E_{i}}{E_{i}}\times 100\%, \end{array}$$

where *E* is the sample of experimental value, *P* is the sample of predicted value by one model, and *N* is the number of strain-stress samples.  Figure 10 shows the relative errors (*η*) of two models depicted as relative frequency versus relative error plot. It can be seen that the constitutive equations are in the range of −39.99%~35.05%; whereas those are found to vary from −3.77% to 16.74% for the ANN model. Meanwhile, the relative error (*η*) within ±1% was observed for more than 79% of predicted data set of the ANN model while only for 16.3% of the predicted data sets for the constitutive equations, which reveals the higher accuracy of the ANN model.

All the results obtained above obviously indicate that the established ANN model showed good performance and could be applied to predict the flow behavior of 20MnNiMo alloys more accurately than the Improved Arrhenius-type models. This is because the response of deformation behaviors of the materials under elevated temperatures and strain-rates is highly nonlinear, and many factors affecting the flow stress are also nonlinear, which make the prediction accuracy of the flow stress by the constitutive equations low and the applicable range limited [31, 32].

### 3.5. Prediction Potentiality of ANN Model Outside the Experimental Condition

**Figure 11:**
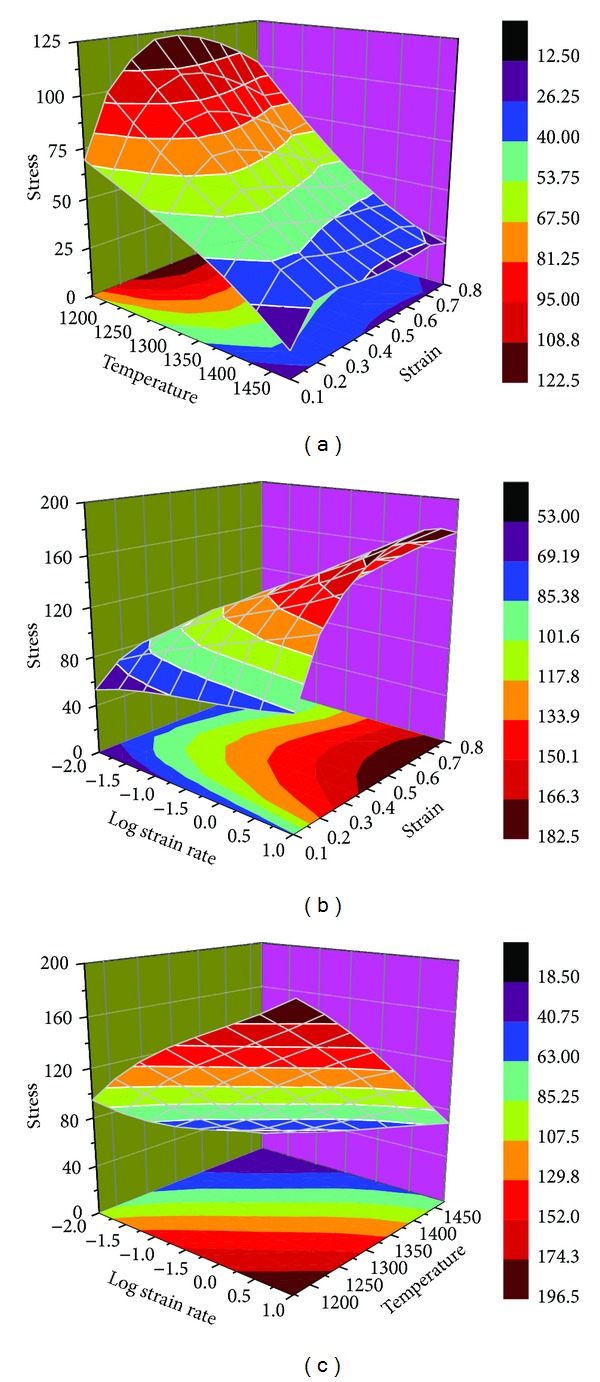
ANN model prediction on the relationship of flow stress versus (a) strain and temperature at strain rate of 0.01 s^−1^ (b) strain and log strain rate at temperature of 1273 K (c) log strain rate and temperature at strain of 0.5.

It is well known that the well-trained ANN models could provide highly accurate prediction of flow stress over a wider range of temperatures and strain rates. Figures 11(a), 11(b), and 11(c) show the 3D surface plots representing the relationships of predicted flow stress versus strain and temperature, strain and log strain rate and log strain rate and temperature, respectively, at a fixed log strain rate of −1.5, temperature of 1223 K, and strain of 0.5. Each node of the surface plots represents a data predicted by ANN model and most of the predicted data were outside the experimental conditions. From Figure 11 it can be seen that the flow stress varies from 0 to 200 MPa with increase in the strain rate from 0.01 to 10 s^−1^ and decrease in temperature from 1173 to 1473 K, which indicates that the predictions are well in agreement with the experimental knowledge.

## 4. Conclusions

The experimental stress-strain data from the isothermal hot compression tests on a Gleeble-1500 thermomechanical simulator, in a wide range of temperatures (1173~1473 K) and strain rates (0.01~10 s^−1^), were employed to develop the Improved Arrhenius-type constitutive model and ANN constitutive model for 20MnNiMo alloys. A comparative study was carried out on their capability to represent the high-temperature deformation behavior of 20MnNiMo alloy. The conclusions can be drawn as follows.

1. The correlation coefficient (*R*) and average absolute relative error (AARE) for the improved Arrhenius-type model are 0.9954 and 5.26%, respectively, while their values for the ANN model are 0.9997 and 1.02%, respectively. Higher *R*-values and lower AARE-values for the ANN model indicate that it has a good predictability under limited experimental conditions.
2. The relative errors (*η*) of the improved Arrhenius-type model and the ANN model were, respectively, in the range of −39.99%~35.05% and −3.77%~16.74%. As for the former, only 16.3% of the test data set possesses *η*-values within ±1%, while, as for the latter, more than 79% possesses. The results indicate that the ANN model presents a higher predictable ability than the improved Arrhenius-type constitutive model.
3. The ANN model for as-cast 20MnNiMo alloy accurately track the experimental data over a wider range of temperatures and strain rates (not only under limited experimental conditions but also outside of experimental conditions). Well-trained ANN models provide fast, accurate, and consistent results, making them superior to the Improved Arrhenius-type constitutive equation. The ANN could also be a good forecast tool to study the high-temperature deformation behavior of other alloys in materials science.

## Figures and Tables

**Table 1:** Coefficients of the polynomial for *Q*, *n*, ln⁡*A*, and *α*.

*Q*		*n*		ln⁡*A*		*α*	
*B*_0_	3.65102	*C*_0_	−0.679	*D*_0_	11.623	*E*_0_	5.15944
*B*_1_	435.05898	*C*_1_	426.108	*D*_1_	5075.860	*E*_1_	−111.124
*B*_2_	−7461.731	*C*_2_	−5647.043	*D*_2_	−83266.642	*E*_2_	1222.173
*B*_3_	58435.579	*C*_3_	36439.007	*D*_3_	636234.990	*E*_3_	−7277.770
*B*_4_	−260734.696	*C*_4_	−138689.722	*D*_4_	−2791840	*E*_4_	26505.195
*B*_5_	712681.632	*C*_5_	331449.121	*D*_5_	7541680	*E*_5_	−61800.962
*B*_6_	−1215160	*C*_6_	−503008.253	*D*_6_	−12749000	*E*_6_	92570.232
*B*_7_	1260820	*C*_7_	470572.854	*D*_7_	13142600	*E*_7_	−86054.036
*B*_8_	−728384.449	*C*_8_	−247492.493	*D*_8_	−7554410	*E*_8_	45145.823
*B*_9_	179648.777	*C*_9_	55999.977	*D*_9_	1855720	*E*_9_	−10212.362
